# On the state-dependent nature of odor perception

**DOI:** 10.3389/fnins.2022.964742

**Published:** 2022-08-26

**Authors:** Laura K. Shanahan, Thorsten Kahnt

**Affiliations:** ^1^Department of Neurology, Feinberg School of Medicine, Northwestern University, Chicago, IL, United States; ^2^Department of Psychology, Rhodes College, Memphis, TN, United States; ^3^National Institute on Drug Abuse Intramural Research Program, Baltimore, MD, United States

**Keywords:** olfaction, olfactory system, odor perception, satiety, circadian rhythms, sleep deprivation, anxiety

## Abstract

The olfactory system—and odor perception by extension—is susceptible to state-dependent influences. This review delves into human behavioral research in this area, and also touches on mechanistic evidence and examples from animal work. The review summarizes studies on the impact of satiety state on olfaction, highlighting the robust effects of food intake on the perceived pleasantness of food odors and olfactory decision-making. The impacts of other behavioral states on olfaction are also discussed. While research in this area is more limited, preliminary evidence suggests that odor perception is altered by circadian state, sleep deprivation, and mood. The flexibility in olfactory function described here can be considered adaptive, as it serves to direct behavior toward stimuli with high state-dependent value.

## Introduction

The sense of smell (i.e., olfaction) is one of the major chemosensory systems, and provides organisms with information about the molecular composition of their immediate environment. All animals possess chemical senses (Yohe and Brand, 2018), including marine animals like *Octopus vulgaris* (Di Cosmo and Polese, 2017; Di Cosmo et al., 2018), and even single-cell organisms like *E. coli* are equipped with receptors that allow them to detect and approach nearby nutrients (Adler, 1969). As such, olfaction informs behaviors that are critical for survival, including food search and predator avoidance (Li and Liberles, 2015), navigation (Raithel and Gottfried, 2021), social interaction (Blomkvist and Hofer, 2021), mate choice (Johansson and Jones, 2007), and caring for offspring (Dulac et al., 2014).

There is not a straightforward mapping between an olfactory stimulus and how to best respond to it. Indeed, the most adaptive response to a given odor can vary depending on the behavioral state of the organism (e.g., how hungry or full they are), so there is adaptive value in modulating olfactory perceptual responses accordingly. In this review, we delve into the state-dependent processing of odors. In particular, we focus on behavioral studies in humans that test the effects of satiety, circadian rhythms, sleep deprivation, and mood on various aspects of olfactory processing. We also incorporate findings from animal studies in this area, and, where available, discuss mechanistic evidence underlying state-dependent olfaction.

## How satiety state impacts olfaction

Appetizing foods exude appetizing smells, which play a substantial role in directing food intake. Thus, there are advantages to satiety state shaping the way we perceive food odors. Anecdotally, on an empty stomach, the delectable scent of a freshly baked apple pie might be more appealing, more intense, more commanding of attention, than it would be in the sated state. This skewed perception can serve to optimize food search and consumption when such cues are behaviorally relevant—i.e., when hungry. There is a substantial body of research on this topic, where the link between satiety and odor pleasantness has been most thoroughly studied.

### Odor pleasantness

As an early example, Michel Cabanac investigated the impact of glucose ingestion on citrus odor perception (Cabanac, 1971). Fasted participants repeatedly rated the scent of orange syrup as pleasant over the course of an hour. When the same participants consumed 100 grams of glucose dissolved in water, their ratings steeply declined in the same timeframe. In his larger body of work, Cabanac observed related trends in temperature and taste perception, and determined that internal bodily signals can dictate whether sensory stimuli are perceived as pleasant or unpleasant (Cabanac, 1979). He coined the term *alliesthesia* to describe this phenomenon (from Ancient Greek words ethesia [meaning sensation] and allios [meaning changed]). Cabanac’s group also found that eating a meal reduced the perceived pleasantness of food odors (e.g., cheese, fish), but not of non-food odors (e.g., lavender, chlorox) (Duclaux et al., 1973). Interestingly, the degree to which satiety triggers olfactory alliesthesia depends on multiple factors, including the concentration of the compounds consumed (Cabanac and Fantino, 1977) and body weight (although it cannot be overlooked that the experimenters themselves acted as participants in the study connecting weight loss to alliesthesia!) (Cabanac et al., 1971).

Related research has explored the concept of sensory-specific satiety—i.e., the ability of food intake to render the sensory properties of foods as less pleasurable across sensory modalities (Rolls, 1986; Hetherington and Rolls, 2004). In a seminal study that demonstrated sensory-specific satiety in the olfactory domain, participants rated the pleasantness of banana, satsuma (a kind of orange), fish, chicken, and rosewater odors (Rolls and Rolls, 1997). Ratings were acquired before and after participants ate an *ad libitum* meal of either bananas or chicken. Participants found the scent of the consumed food to be less pleasant after the meal, but ratings for the other odors did not change. These results extend those from the Cabanac studies by revealing an additional layer of specificity. Namely, satiety alters the way we perceive the scent of *specific* food items that were recently consumed.

The result that participants find food odors less appealing after they have eaten the food in question has been replicated many times (O’Doherty et al., 2000; Hollis and Henry, 2006; Brondel et al., 2007; Fernandez et al., 2013; Howard and Gottfried, 2014; Stafford, 2016; Howard and Kahnt, 2017; Howard et al., 2020). Studies along these lines have used natural food odors and synthetic food odors, presented manually or *via* computer-controlled olfactometers. Although most of these studies were carried out in a lab environment, the effect has also been demonstrated in a restaurant setting using aromatized appetizers and desserts (Fernandez et al., 2013). Thus, the effect is robust and generalizable across different study designs.

Cabanac’s alliesthesia experiments highlight the role of internal signals in modulating food odor perception since they involved ingestion of a glucose solution, which does not exhibit the complex sensory features of a legitimate meal. Moreover, the effect manifested whether the glucose solution was ingested orally or injected into the stomach directly via a nasogastric tube (Cabanac and Fantino, 1977), indicating that it is not driven by sensory interaction with the solution. In contrast, sensory-specific satiety studies emphasize the importance of the food’s sensory features. In the chicken and banana experiment described earlier, meal ingestion was not the only way to induce olfactory sensory-specific satiety. A similar (albeit smaller) effect was observed when participants simply chewed their food without swallowing it, or when they were exposed to the smell of the food for 5 min (Rolls and Rolls, 1997), which suggests that digestive mechanisms cannot be solely responsible. This is perhaps unsurprising given the complex cross-modal interactions between olfaction and other sensory modalities (Morrot and Dubourdieu, 2001; Dematté et al., 2006), which require integration across sensory systems.

To explore the brain mechanisms underlying these state-dependent effects, O’Doherty et al. (2000) used functional magnetic resonance imaging (fMRI). Participants smelled banana and vanilla odors during fMRI scanning, both before and after eating a meal of bananas. They found that odors evoked brain activity in orbitofrontal cortex (OFC), which is consistent with other olfactory neuroscience research (Zatorre et al., 1992; Gottfried and Zald, 2005). OFC activity evoked by banana odor declined after the meal along with pleasantness ratings, implicating this brain region in olfactory sensory-specific satiety. Although this experiment was conducted in a small sample (*n* = 5), the results are compatible with primate electrophysiology work showing that OFC neurons are less responsive to a food odor after consuming an odor-matched meal (Critchley and Rolls, 1996). Taken together, this work suggests that odor pleasantness is shaped by nutritional context through a combination of enteric, sensory, and higher-order neural signals.

### Odor sensitivity

Food consumption clearly modulates food odor pleasantness, but is odor sensitivity also affected? In an early attempt to address this question, John Glaze used a device called the Zwaardemaker olfactometer to present odors to participants at a range of concentrations by physically manipulating the distance between a given odor and the participant’s nostrils (Glaze, 1928). He found that two participants (Glaze himself and a 10-year-old boy attempting to lose weight) became more sensitive to a collection of unique odors (e.g., cedar wood, rubber, beeswax, Russian leather) over the course of a 5-day fast. In a follow-up experiment, Glaze measured participants’ sensitivity to the same odors before and after lunch. The participants were more sensitive to odors before lunch, and Glaze concluded that olfactory sensitivity is markedly enhanced in the fasted state.

Several other studies in the mid-twentieth century attempted to quantify the effects of satiety state on olfactory sensitivity (Janowitz and Grossman, 1949; Goetzl et al., 1950; Hammer, 1951; Schneider and Wolf, 1955; Zilstorff-Pedersen, 1955; Guild, 1956; Furchtgott and Friedman, 1960; Turner and Patterson, 1966), often by exposing participants to the scent of coffee before and after a midday meal. More recent studies typically use a collection of Sniffin’ Sticks threshold pens (Burghardt, Wendel, Germany) to assess odor sensitivity (Hummel et al., 1997) across hungry and sated states (Schreder et al., 2008; Albrecht et al., 2009; Stafford and Welbeck, 2011; Enck et al., 2014; Hanci and Altun, 2016). To administer this test, experimenters present pens containing varying concentrations of a target odor (e.g., n-butanol) together with foil pens (containing only solvent) to participants’ noses in a systematic sequence, prompting them to identify the target pen. Unfortunately, results from the earlier studies, as well as Sniffin’ Sticks studies, are very mixed. In contrast, findings from a handful of rodent studies are more consistent, and suggest enhanced odor sensitivity in the hungry state (Aimé et al., 2007, 2014; Prud’homme et al., 2009), with potential mechanistic ties to olfactory bulb activity (Prud’homme et al., 2009), and fluctuations in hormones and neuromodulators (Julliard et al., 2007; Tong et al., 2011; Soria-Gómez et al., 2014).

As such, the relationship between odor sensitivity and hunger state remains an open question, at least in humans. It is noteworthy that coffee and n-butanol are the most common odors tested in these studies. Coffee was likely selected because it was described as a pure olfactory stimulus (i.e., with no trigeminal component) in the mid-1930’s (Elsberg et al., 1935), whereas n-butanol is one of the default odors in the Sniffin’ Sticks threshold test. Indeed, very few studies have tested sensitivity to food odors, leaving open the possibility that food intake might have more consistent effects on food odor sensitivity.

To address this gap, Ramaekers et al. (2016) strategically tested sensitivity to vanillin and meat broth odors after participants ate a sweet lunch, a savory lunch. The food items closely matched the odors, with the sweet lunch containing vanilla custard and the savory lunch containing chicken noodle soup. The experimenters found that participants were more sensitive to food odors in the hungry state, regardless of whether the sweet or savory lunch was consumed. These effects were nominally larger following the odor-matched meal, though not significantly so. This study provides strong evidence that satiety state reduces sensitivity to food odors, but more studies using food odors as stimuli are needed to further establish this relationship.

### Olfactory perceptual decision-making

**FIGURE 1:**
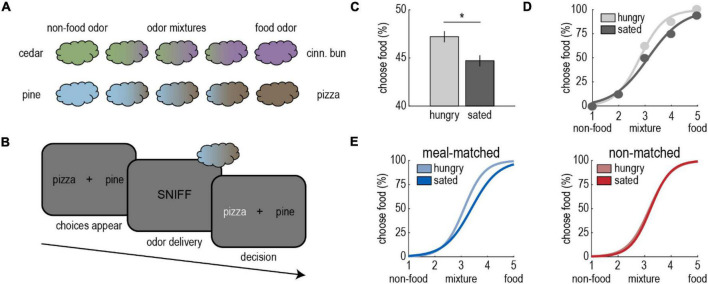
Satiety state modulates olfactory perceptual decision-making. **(A)** Olfactory stimuli included cedar, cinnamon bun, pine, and pizza odors, as well as non-food/food odor mixtures (cedar/cinnamon bun, pine/pizza). **(B)** On each trial of the olfactory decision-making task, participants indicated whether they perceived the odor to be food-dominant or non-food-dominant. They completed the task in the hungry state, and then again after a meal of cinnamon buns or pizza. **(C)** Participants were more likely to perceive odors as food-dominant in the hungry state. **(D)** Sigmoidal functions were fitted to each participants’ choice data for meal-matched and non-matched odor pairs separately. The figure depicts a single participant’s average perceptual choices and fitted choice curves for the meal-matched odor pair. **(E)** After the meal, participants’ perceptual choice curves shifted toward the food odor, but only for the meal-matched odor pair. In more specific terms, after eating a meal of cinnamon buns, more cinnamon bun odor was required in cedar/cinnamon bun mixtures for participants to perceive them as food-dominant, while pine/pizza mixtures were unaffected, and the opposite was true after a meal of pizza. Adapted from Shanahan et al. (2021).

To build on these findings, our lab investigated the impact of food intake on perceptual decision-making—a function that is frequently studied in the visual domain (e.g., Britten et al., 1992). To accomplish this, we designed a novel olfactory task where perception of food odors was pitted directly against that of non-food odors (Figure 1A; Shanahan et al., 2021). On each trial, participants smelled odor mixtures containing either cedar and cinnamon bun or pine and pizza, and they chose the component they perceived as dominant in the mixture (Figure 1B). They performed the task before and after an odor-matched meal of cinnamon buns or pizza.

We found that participants were more likely to perceive mixtures as food-dominant in the hungry state (Figure 1C). Intriguingly, this effect was driven by the meal-matched odor (Figures 1D,E), and behavioral changes were paralleled by changes in fMRI activity in piriform cortex and amygdala. These results lend further credibility to the idea that food odor sensitivity is enhanced in the hungry state, and suggest specificity akin to the sensory-specific effects observed in studies of odor pleasantness.

## How other behavioral states impact olfaction

There are a number of behavioral states that influence odor perception besides satiety. Although these relationships are less commonly studied, we review some initial findings in the following sections.

### Circadian state

A handful of studies suggest that odor perception may fluctuate based on time of day (Goetzl et al., 1950) or, relatedly, circadian state (Nordin et al., 2003; Herz et al., 2017). In the most recent of these, participants underwent a forced desynchrony protocol, where they followed a sleep/wake schedule consistent with a 28-h day for over a week (Herz et al., 2017). Since the circadian clock stays true to its 24-h cycle during forced desynchronization (Kleitman, 1939; Dijk and Czeisler, 1994), this method is used extensively in circadian research to disentangle circadian timing from time of day effects. The experimenters administered Sniffin’ Sticks threshold tests containing phenylethyl alcohol (PEA; rose scent) at six time points each cycle. They found that scores fluctuated with circadian phase, such that sensitivity peaked slightly after melatonin production onset, corresponding to around 9:00 pm for the usual 24-h cycle. The authors speculated that this olfactory circadian peak may have evolved to facilitate detection of predators after dark, meal satisfaction, and mate selection. While additional evidence is needed to confirm and build upon these findings, they parallel results in *Drosophila*, where the circadian nature of olfactory responses is well-documented, and depends on known circadian clock genes (Krishnan et al., 1999, 2001; Tanoue et al., 2004; Hardin, 2005).

### Sleep deprivation

Relatedly, olfaction may be influenced by sleep deprivation. One group tested participants’ performance on the Smell Identification Test (SIT) before and after 24 h of sleep deprivation (Killgore and McBride, 2006). The SIT consists of a test booklet containing common odors (e.g., chocolate, rose, soap, smoke), which participants must identify from four multiple choice options (Doty et al., 1984). Participants performed significantly worse on the SIT in the sleep-deprived state. In a follow-up study, SIT scores declined significantly, and to a similar degree, following 52 h of sleep deprivation (McBride et al., 2006). The authors speculated that compromised olfactory performance could be related to OFC function, since an earlier positron emission tomography study demonstrated a relative decrease in OFC activity in the sleep-deprived state (Thomas et al., 2000). An important caveat of these studies is that, in addition to intact olfactory function, the SIT requires other cognitive functions, such as language and object recognition, which could be separately impacted by sleep deprivation.

Since the sleep-deprived state has been linked to unhealthy food choices (Nedeltcheva et al., 2009; Markwald et al., 2013), food odor perception might be particularly impacted. Our lab set out to address this question by exposing participants to food and non-food odors during two fMRI scanning sessions – one after a full night of sleep, and one following a night of partial sleep deprivation (in a counterbalanced order) (Bhutani et al., 2019). After fMRI scanning, participants were invited to consume a selection of sweet and savory food items in a buffet-style setting. Findings indicated that sleep deprivation sharpens neural responses evoked by food odors in piriform cortex and shifts food choices toward items that are more calorie-dense.

Altogether, these studies suggest a relationship between sleep deprivation and odor perception, but far more research is needed to further characterize this connection. It is also worth noting that odor perception and olfactory brain activity are altered *during* sleep (Carskadon and Herz, 2004; Barnes and Wilson, 2014), but a full account of this work is beyond the scope of our review.

### Mood

Beyond circadian and sleep-related states, olfactory function has been shown to vary with emotional states, most notably anxiety. Three recent studies suggest that anxiety modulates odor perception (Pacharra et al., 2016; Hoenen et al., 2017; Cortese et al., 2021). All of these implemented some version of the Trier Social Stress Test (TSST). The TSST requires participants to engage in various stressful activities in front of experimenters, such as mental arithmetic or presenting a speech, to induce stress and anxiety in the lab. One such study demonstrated that a TSST-induced increase in cortisol was significantly associated with better SIT performance, and an increase in perceived odor intensity (Hoenen et al., 2017). The other two studies measured odor thresholds. In one of them, participants were shown to be more sensitive to a foul odor (2-mercaptoethanol, which smells like sewage) in the anxious state (Pacharra et al., 2016). In the other, participants with high anxiety severity were shown to be more sensitive to the scent of smoke (guaicol) than the scent of rose (PEA), when compared to participants with low anxiety severity (Cortese et al., 2021). Although the latter result speaks more to trait anxiety than state anxiety, this relationship was further accentuated by the TSST.

Along similar lines, mood induction has been shown to influence odor perception, with participants rating odors as less pleasant following a negative mood induction and more pleasant following a positive mood induction (Pollatos et al., 2007). Although, once again, more studies in this area are needed to better understand how mood interacts with odor perception, these findings suggest that such a relationship exists, and that effects may vary depending on the characteristics of the odor in question.

## Conclusion

In this review, we summarize the literature on state-dependent olfaction in humans. Most of this work centers on satiety state. Food odors are perceived as more pleasant, and perhaps more salient, when hungry, allowing olfaction to guide food search when nutrients are needed most. In many cases, these satiety state-dependent effects are specific to food odors that closely match the consumed food, which may help to regulate intake of specific foods and facilitate nutritional balance. Preliminary evidence suggests that other behavioral states, such as circadian state, sleep deprivation, and mood, also modulate odor perception, although these effects are not nearly as established as they are in the case of satiety state. While behavioral studies are most prevalent in the human literature, and are thus the primary focus of this review, emerging mechanistic work highlights the role of primary and secondary olfactory cortices in state-dependent olfactory processing.

Taken together, the work described here shows clear evidence for the state-dependent nature of olfaction. However, there is substantial overlap between the behavioral states discussed. For instance, hunger typically manifests at particular times of day, anxiety increases with sleep deprivation, and mood fluctuates with hunger state. Future state-dependent olfactory research should seek to characterize individual contributions of these behavioral states more fully, while also illuminating common themes across states. This represents an opportunity to implement new and creative olfactory behavioral tests, and ideally to coordinate odor selection across studies. For instance, implementing food odors in studies of circadian state, sleep deprivation, and mood could reveal hidden links to satiety-related findings. By continuing to investigate odor perception through the adaptive lens of behavioral state, we can gain critical insights into olfactory processing, as well as the broader connections between olfaction, health, and well-being.

## Author contributions

Both authors conceptualized and wrote the manuscript and approved the submitted version.
